# Editorial: Challenges in fertilization and implantation success

**DOI:** 10.3389/fcell.2023.1268635

**Published:** 2023-08-30

**Authors:** Claudia Massarotti, Sofia Makieva, Sara Stigliani

**Affiliations:** ^1^ Academic Unit of Obstetrics and Gynecology, Maternal-Child Department, IRCCS Ospedale Policlinico San Martino, Genoa, Italy; ^2^ Department of Neurosciences, Rehabilitation, Ophthalmology, Genetics and Maternal and Child Health (DINOGMI Department), University of Genoa, Genoa, Italy; ^3^ Department of Reproductive Endocrinology, University Hospital Zurich, Zurich, Switzerland; ^4^ Physiopathology of Human Reproduction Unit, IRCCS Ospedale Policlinico San Martino, Genoa, Italy

**Keywords:** IVF, fertilization (IVF), human reproduction, implantation, repeated implantation failure (RIF), endometriosis, extracellular vehicles (EVs)

Ever since the first successful birth through *in vitro* fertilization (IVF) in July 1978, the utilization of assisted reproduction technologies (ART) has steadily risen, resulting in the birth of over 10 million babies. However, despite advancements in technology and ongoing research endeavours, the success rates have seen little improvement over the past decade, with a live birth rate of approximately 30% per cycle (European IVF Monitoring Consortium et al., 2022). While we have achieved the ability to replicate fertilization entirely in a laboratory environment, we still lack a comprehensive understanding of the intricate biological processes involved in the interaction between oocytes and sperm that lead to a successful embryo development. We also do not fully comprehend the pathophysiology of implantation and the establishment of a successful ART pregnancy. The aim of this Research Topic was to further our understanding of the very first steps of human life, identifying possible promising biological mechanisms to study, pondering the influence of various factors in clinical practice and identifying the role of possible interventions. We purposefully welcomed contributions from basic to translational to clinical research, because we believe that only by crossing bridges from the laboratory to the clinic will we have the ability to answer challenging questions.

Two review articles with a basic science background highlighted the role of microRNAs, which are commonly packaged in nanosized vesicles surrounded by a lipid bilayer called extracellular vesicles (EVs). EVs are secreted by cells into the extracellular space to facilitate cell-to-cell communication. Fan et al. reviewed the roles of the EVs secreted by oocytes, granulosa cells and cumulus cells, and narrated their messenger functions in oocyte development, fertilization, protection of sperm, the acrosome reaction, early embryo development and in the communication between embryo and the endometrium during implantation. Many questions about their formation and secretion, mechanism of action and actual role in humans (most studies are in an animal model) are however still unanswered and a better understanding of their role could be of use also to optimize ART techniques success.

Taraschi et al.’s work provides collective evidence on the oviductal miRNome and contributes to the ongoing efforts to elucidate the functions of EVs in animal reproduction. Specifically, in that review the authors performed a comprehensive assessment of the microRNA landscape in the oviductal fluid. They described the oviductal miRNAome from omics data through gene autology enrichment analysis and explored the role of every protein coding gene with knockout mouse lines, using the IMFC database. They found genes with a role in fertilization and early embryo development, confirming the role of the miRNA transported by EVs in the early stages of reproduction.

Moving on to the clinical setting, we featured studies dealing with factors influencing gametes (and embryo) quality, such as patients’ characteristics, age, and the impact of chronic diseases/therapies.

Sachs et al. reported on ART results in women with endometriosis. They compared the outcome of 66 frozen-warmed unbiopsied single blastocyst transfers of patients with endometriosis to 96 controls (women with idiopathic infertility). They found no impairment in the ovarian stimulation response, not even in women with an endometrioma, but a higher miscarriage rate. This finding, coherent with the data available in literature, points to a defective implantation process. Possible causes are the presence of a highly inflammatory microenvironment that alters endometrium receptivity or a concomitant adenomyosis, but a role of a diminished oocyte/embryo quality cannot be excluded.

In order to investigate the potential influence of laboratory conditions on embryo development and successful implantation, Makieva et al. conducted a retrospective study examining the outcomes of frozen embryo transfer (FET) using previously cryopreserved zygotes, categorized based on the time of freezing. They found higher pregnancy rates in embryo transfers where vitrification was realized after 19:01 h post-insemination (hpi) and before 21:00 hpi compared to earlier than 18:00 hpi in the univariate analysis. However, the chances of pregnancy seemed not influenced any more by the precise timing (provided it was within the 17 to 21 hpi window recommended for 2PN stage vitrification) when all relevant factors were considered in a multivariate analysis. The authors reflected on the biological rationale behind the standard timing: the S phase, during which zygotes are susceptible to damage and should never be exposed to cryopreservation, is expected to end between 10 and 18 hpi. They hypothesize that some of the zygotes cryopreserved earlier may have not ended this delicate phase yet, explaining worse results. Only a better understanding of early embryo development may give definitive answers.

Zhu et al. performed a meta-analysis of 15 RCTs (4,281 participants) to explore the beneficial effect of transcutaneous electrical acupoint stimulation (TEAS) on ART outcomes. They found increased implantation, pregnancy and live birth rates in cases compared to controls, however these results have to be interpreted with caution since the original studies had a low numerosity, high heterogeneity and different protocols were used.

The successful progression of pregnancy heavily relies on a delicate harmony between a properly developing embryo and the receptive endometrium. Despite considerable research on the subject of failed implantation and some proposed methods for assessing endometrial receptivity, significant controversies persist, starting with the existence and accurate definition of repeated failed implantation (RIF). Li et al. explored the possible role of chronic endometritis, to investigate if women with Mum1+/CD138+ plasma cells at the endometrial biopsy showed lower rates of implantation and pregnancy. The hypothesis that an inflamed endometrium may be less receptive has rationale, but several key questions remain unanswered regarding the definition, diagnosis, and appropriate treatment of chronic endometritis. The authors performed an endometrial biopsy on 327 RIF patients and then treated the positive patients with antibiotics and, in case the biopsy was persistently positive, with platelet rich plasma (PRP) before proceeding to embryo transfer. They found a high prevalence of chronic endometritis in these patients (35.78%), but the majority of them (70% of positive cases) converted to negative after treatment. The researchers then compared the outcomes of frozen embryo transfers in three groups: patients who consistently tested negative, those who became negative after treatment, and those who still showed weak positivity even after treatment. The results indicated that the group with persistent weak positivity had decreased rates of implantation, pregnancy, and live births. While these findings are intriguing, it is essential to note that before implementing experimental treatments like PRP in clinical practice, further investigation of its efficacy and safety through well-designed randomized controlled trials is necessary. This will provide a more robust understanding of the potential benefits and risks associated with such therapies.

Continuing with obstetric complications associated with the intricate processes of implantation and placentation, Mei et al. reported their experience with subchorionic hematoma, a partial detachment of the chorion membranes from the wall of the uterus, in twin pregnancy obtained with ART. Approximately 38% of patients developed SCH in the first trimester. Independent risk factors for SCH included male factor, hydrosalpinx, polycystic ovary syndrome (PCOS), previous miscarriage, and adenomyosis. The SCH group showed a significantly higher rate of twin pregnancy loss before 20 gestational weeks. The study emphasizes the importance of increased surveillance and timely treatment for patients with SCH to improve pregnancy outcomes.

Finally, to see the discussed Research Topic from another broader perspective, Li Piani et al. narrative review explored the potential links between epigenetic aging and the fertility timeline. The authors delved into advances in epigenetic clocks, focusing on various tissues examined in infertility cases, such as the endometrium, peripheral blood, and ovaries. Additionally, it discusses findings concerning epigenetic aging during pregnancy. They concluded that epigenetic clocks may represent a promising research tool to bring us closer to understanding the complexities of reproductive function, including the aspects of fertilization and implantation that remain elusive. Understanding the role of epigenetic aging in infertility mechanisms and pregnancy outcomes could unveil the epigenetic clock as a connecting factor between seemingly contrasting worlds: infertility and pregnancy.

The substantial number of articles submitted to this Research Topic serves as compelling evidence for the enduring interest and vibrant scientific engagement surrounding the intricate processes of fertilization and implantation. Despite the progress made thus far, numerous unanswered questions persist, underscoring the need for a comprehensive integration of basic, translational, and clinical research. It is through this collaborative approach that we hope to unravel the enigmatic aspects of human reproduction that still elude our understanding.

## References

[B1] European IVF Monitoring Consortium for the European Society of Human Reproduction and Embryology Wyns C. De Geyter C. Calhaz-Jorge C. Kupka M. S. Motrenko T. Smeenk J. (2022). ART in europe, 2018: results generated from European registries by ESHRE. Hum. Reprod. Open 2022 (3), hoac022. 10.1093/hropen/hoac022 35795850 PMC9252765

